# Pooled analysis of menstrual irregularities from three major clinical studies evaluating everolimus for the treatment of tuberous sclerosis complex

**DOI:** 10.1371/journal.pone.0186235

**Published:** 2017-10-12

**Authors:** Steven Sparagana, David N. Franz, Darcy A. Krueger, John J. Bissler, Noah Berkowitz, Karin Burock, J. Christopher Kingswood

**Affiliations:** 1 Texas Scottish Rite Hospital for Children and University of Texas Southwestern Medical Center, Dallas, TX, United States of America; 2 Cincinnati Children’s Hospital Medical Center, Cincinnati, OH, United States of America; 3 St. Jude Children’s Research Hospital and Le Bonheur Children’s Hospital, Memphis, TN, United States of America; 4 Novartis Pharmaceuticals Corporation, East Hanover, NJ, United States of America; 5 Novartis Pharma AG, Basel, Switzerland; 6 Royal Sussex County Hospital, Brighton, United Kingdom; Vanderbilt University Medical Center, UNITED STATES

## Abstract

**Objectives:**

To determine the impact of everolimus on female fertility, including menstrual irregularities, secondary amenorrhea, and luteinizing and follicle stimulating hormone levels in female patients.

**Design:**

A pooled analysis from 3 prospective studies consisting of a core phase (≥6 months) and a long-term follow-up open-label extension.

**Setting:**

One phase 2 single-center and two phase 3 multicenter studies.

**Participants:**

Data were obtained from female participants, restricted to those between 10 and 55 years of age, during 1 of 3 of the described clinical trials of everolimus. Patients had received ≥ 1 dose of everolimus.

**Main outcome measures:**

Incidence of fertility events.

**Results:**

A total of 43/112 patients (38.4%) experienced at least 1 menstrual irregularity. The most common events were amenorrhea (24.1%) and irregular menstruation (17.0%). Seven patients (6.3%) experienced grade 3/4 amenorrhea. When only the longest duration period of amenorrhea for each patient was considered, the median duration was 291 days. Fifteen patients attained menarche during the treatment period in any of the pooled studies. The mean age of menarche for this group was 12.4 years, similar to that of patients who were postmenarche at study entry (12.2 years). A total of 19/92 patients (20.7%) who were postmenarche at baseline or during the study experienced an irregular menstruation event. An increased luteinizing hormone level was reported as an adverse event in 3/112 patients (3%), and follicle-stimulating hormone levels were within normal limits for these patients.

**Conclusions:**

No new safety concerns emerged regarding endocrine function and menstruation in female patients with tuberous sclerosis complex–associated subependymal giant cell astrocytoma or angiomyolipoma, who were receiving everolimus.

**Trial registration:**

**ClinicalTrials.gov** NCT00411619, NCT00789828, NCT00790400

## Introduction

Tuberous sclerosis complex (TSC) is a multisystemic, autosomal dominant genetic disorder that occurs in approximately 1:6000 births [1] and affects up to 1 million people globally [2]. This disorder is a result of mutations in the *TSC1* or *TSC2* genes, responsible for producing protein complexes that act on the mammalian target of rapamycin (mTOR) complex, mTORC1 [3–8]. TSC deficiency, as a consequence, leads to the upregulation of the mTORC1 complex with downstream effects including abnormal cellular growth, proliferation, and protein synthesis [3,9]. Common physical manifestations of TSC include hamartomas, cortical tubers, subependymal giant cell astrocytomas (SEGAs) and subependymal nodules in the brain, angiomyolipomas in the kidneys, and lymphangioleiomyomatosis in the lungs [9].

The involvement of the mTOR complex in TSC has made it a relevant target of investigation for TSC therapies, with a focus on the development of mTOR inhibitors. Several studies have demonstrated improvements in TSC-associated symptoms including SEGAs, angiomyolipomas, and facial angiofibromas in response to mTOR inhibitors [10–13]. Everolimus is an mTOR inhibitor that has been approved for treatment in adults with renal angiomyolipomas and TSC not requiring immediate surgery. It has also been approved in pediatric and adult patients with TSC who have SEGAs that require therapeutic intervention but cannot be curatively resected [14].

The mTOR signaling pathway has also been suggested to play a potential role in reproductive processes including the onset of puberty [15]. Studies have reported that activation of mTOR in female pubertal rats was linked to defective gonadotropin levels and delayed puberty [16]. Furthermore, a blockade in the mTORC1 signaling cascade in prepubertal female rats, using the mTOR inhibitor rapamycin, has been shown to partially inhibit the onset of puberty [17]. In clinical trials, menstrual irregularities, secondary amenorrhea, and increases in luteinizing hormone (LH) and follicle-stimulating hormone (FSH) have been observed in female patients taking everolimus [2,10,18,19]. Based on these clinical findings, and findings in preclinical safety studies, further study is required regarding the impact of everolimus on female fertility.

To further quantify these potential effects on female fertility, we conducted a pooled analysis of menstrual irregularities, including secondary amenorrhea, restricted to patients who were between 10 and 55 years of age during participation in 1 of 3 clinical trials of everolimus in patients with TSC.

## Methods

### Study design

**Fig 1:**
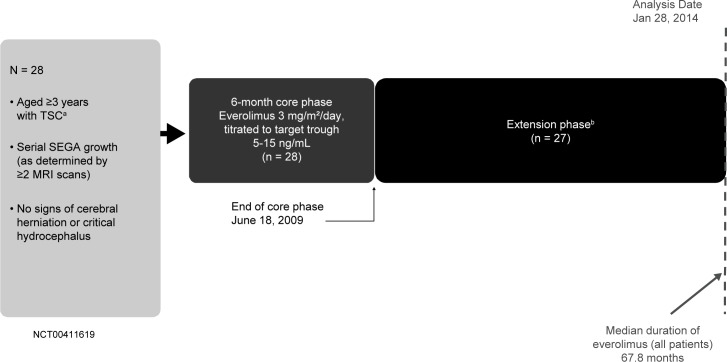
Study design of the phase 2 study. MRI, magnetic resonance imaging; SEGA, subependymal giant cell astrocytoma; TSC, tuberous sclerosis complex. ^a^Definite diagnosis per modified Gomez criteria or a positive genetic test. ^b^Upon completion of the core phase, patients could continue to receive everolimus if evidence of therapeutic benefit.

**Fig 2:**
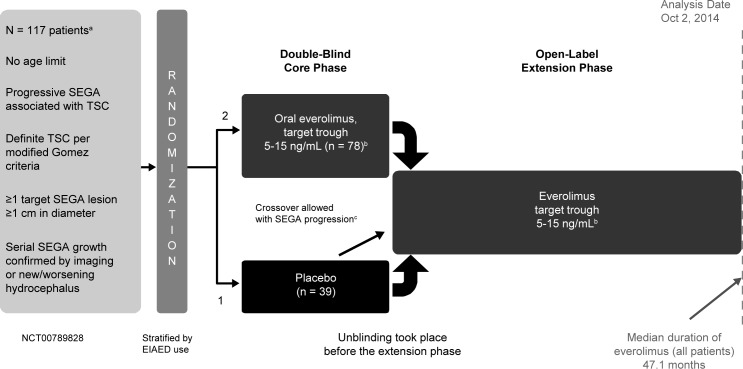
Study design of EXIST-1. EIAED, enzyme-inducing antiepileptic drug; SEGA, subependymal giant cell astrocytoma; TSC, tuberous sclerosis complex. ^a^Enrollment occurred between August 20, 2009, and September 2, 2010. ^b^Everolimus starting dose: 4.5 mg/m^2^/day, adjusted to blood trough levels of 5–15 ng/mL. Dose could be adjusted in cases of toxicity. ^c^SEGA progression was defined as an increase in SEGA volume ≥ 25% from lowest value to a value greater than baseline or as appearance of new SEGA lesions ≥ 1 cm in longest diameter, worsening of nontarget lesions, or new or worsening hydrocephalus.

**Fig 3:**
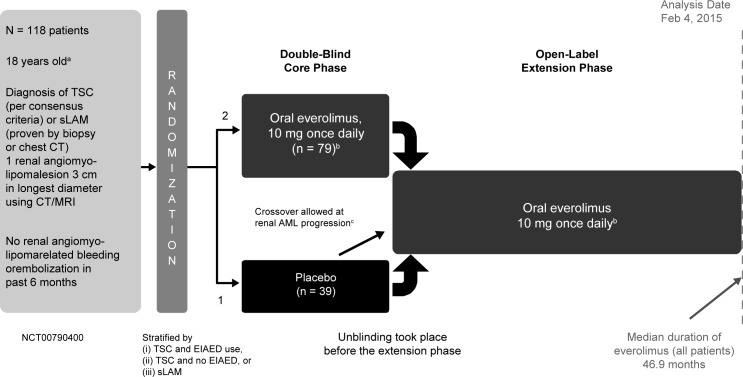
Study design of EXIST-2. AML, angiomyolipoma; CT, computed tomography; EIAED, enzyme-inducing antiepileptic drug; MRI, magnetic resonance imaging; sLAM, sporadic lymphangioleiomyomatosis; TSC, tuberous sclerosis complex. ^a^Enrollment occurred between April 28, 2009, and December 30, 2010. ^b^Dose adjusted based on toxicity. ^c^Renal angiomyolipoma progression by central review or occurrence of adverse event of angiomyolipoma-related bleeding grade 2 or worse.

Data were combined from 3 studies evaluating the use of everolimus in treating TSC-associated SEGA or renal angiomyolipoma. A prospective, open-label, single-arm, single-center, phase 2 study (NCT00411619) [20] and 2 prospective, double-blind, randomized, parallel-group, placebo-controlled, multicenter, phase 3 studies (EXIST-1 [NCT00789828] and EXIST-2 [NCT00790400]) [10,18]. The study protocols were approved by ethics committees at each center before the first patient was enrolled (S1 Table provides a list of IRBs for each study). These studies were conducted in accordance with the principles of Good Clinical Practice, Declaration of Helsinki, and all local regulations. All patients (or their legal representatives) provided written informed consent before enrollment. All 3 studies consisted of a core phase (≥6 months) followed by a long-term open-label extension (Fig 1, Fig 2 and Fig 3). Patients were to be followed up for 4–5 years after the last patient was enrolled. In the phase 2 study, the starting dose of everolimus was 3.0 mg/m^2^/day titrated to attain blood trough levels of 5–15 ng/mL. In EXIST-1, the starting dose of everolimus was 4.5 mg/m^2^/day titrated to attain blood trough levels of 5–15 ng/mL. In EXIST-2, the starting dose of everolimus was 10 mg/day. In all trials, further adjustments of dosage were allowed in cases of toxic effects.

### End points

Patient-level data was searched to identify amenorrhea and related events. The definitions of amenorrhea and related events were based on MedDRA (version 17.1) where the standardized medDRA Query (SMQ) term was “fertility disorders,” the higher level term “female gonadal function disorder,” and the preferred terms related to abnormal hormone level. The National Cancer Institute Common Terminology Criteria for Adverse Events (NCI-CTCAE) version 4.0 was used to assess severity of events. NCI-CTCAE defines grades 1, 2, and 3 of irregular menses as 1–3 months without menses, > 3–6 months without menses, and > 6 months without menses (amenorrhea), respectively.

### Statistical analysis

The pooled data set included female patients who received at least 1 dose of everolimus and were between the ages of 10 and 55 years at any point during treatment. Demographics and patient characteristics, female fertility events, and hormone levels were summarized using descriptive statistics. Incidence of events was summarized for the placebo-controlled double-blind phases (combined for EXIST-1 and EXIST-2), and for the combined double-blind and open-label phases of EXIST-1, EXIST-2, and the phase 2 studies. Comparisons were made between treatment groups (i.e., everolimus versus placebo) during the double-blind phases of the EXIST-1 and EXIST-2 trials; this data was pooled, stratified by study and menarche status at baseline, and reported as an odds ratio (OR) with 95% confidence intervals (CIs). A conditional exact Mantel-Haenszel test was used for the odds-ratio estimate of everolimus vs placebo. Crude incidence rates were reported for the overall patient population by study and menarche status at baseline. Time-to-onset of female fertility events was defined as the time from the start of everolimus to the date of the first occurrence of any event (including secondary amenorrhea) of any grade in the subset of patients who were post-menarche at baseline. The Kaplan-Meier method was used to estimate the median time-to-onset of female fertility events and 95% CIs. Data were analyzed using SAS version 9.4.

## Results

### Patients

A total of 119 female patients received everolimus or placebo in the core or the open-label phase, across the 3 studies (Table 1). A total of 112 of these patients were between the ages of 10 and 55 years (at any point in time during study), and received ≥ 1 dose of everolimus in either the core or the open-label phase of the studies. These patients are included in the analysis. Combined duration of everolimus exposure was 418 patient-treatment years. More than half of the patients (n = 72, 64.3%) in this analysis had an everolimus exposure ≥ 3.5 years, and 53 patients (47.3%) were on everolimus for 4 years or more. One patient (0.9%) was on everolimus treatment for 6.5 years.

**Table 1:** Baseline patient demographics and clinical characteristics of at-risk female patients across 3 trials.

N (%)^a^	Pooled Population N = 119
**Age, median (range), years**	23.0 (4–54)
** < 10 years**	16 (13.4)
** 10 to < 20 years**	30 (25.2)
** 20 to < 46 years**	65 (54.6)
** 46 to ≤ 55 years**	8 (6.7)
**Menarche status** **Premenarche** **Postmenarche** **Postmenopausal/no menses**	27 (22.7) 84 (70.6) 8 (6.7)
**Age at menarche, mean (SD), years**^**b**^	12.3 (1.7)

SD, standard deviation.

^a^Unless otherwise specified.

^b^n = 75 because of missing information.

### Female fertility events

A total of 43/112 patients (38.4%; 95% confidence interval [CI], 29.4–48.1) experienced at least 1 event, with a total of 55 menstrually related events (all grades) reported. The most common events were amenorrhea (24.1%) and irregular menstruation (17.0%), mostly of grade 1 or 2 severity (Table 2). Seven patients (6.3%) experienced grade 3/4 amenorrhea. Other reported menstruation- or fertility-related events (all grades) included increased LH level, delayed menstruation, oligomenorrhea, increased blood testosterone, and polycystic ovaries (3, 2, 2, 1, and 1 patient[s], respectively) (Table 2).

**Table 2:** Individuals with menstrual irregularities while on everolimus.

n (%)^a^	Everolimus n = 112	
	Any Grade	Grade 3–4
**Patients with ≥ 1 event** **[95% CI]**	43 (38.4) [29.4–48.1]	7 (6.3) [2.5–12.5]
**Amenorrhea**	27 (24.1)	7 (6.3)
**Irregular menstruation**	19 (17.0)	0
**Increased blood LH level**	3 (2.7)	0
**Menstruation delayed**	2 (1.8)	0
**Oligomenorrhea**	2 (1.8)	0
**Increased blood testosterone**	1 (0.9)^b^	0
**Polycystic ovaries**	1 (0.9)	0

CI, confidence interval; LH, luteinizing hormone.

^a^Unless otherwise specified.

^b^Occurred in a postmenopausal patient.

For comparison, during the placebo-controlled, double-blind phases of EXIST-1 and EXIST-2, 18 of 73 combined patients (24.7%; 95% CI [15.3, 36.1]) who received everolimus experienced at least 1 menstruation- or fertility-related event, versus 1 of 36 patients on placebo (2.8%; 95% CI [0.1, 14.5]). The odds ratio was 8.3 (95% CI [1.0, 66.5]). All were grade 1/2 with the exception of grade 3/4 amenorrhea occurring in 5 patients (6.8%) on everolimus.

Fifteen of the 112 total patients attained menarche during the treatment period in any of the pooled studies. The mean (standard deviation [SD]) age of onset of menarche for these patients was 12.4 (1.0) years, which was similar to the age of onset for the patients who were postmenarche at study entry (n = 57 with known age at menarche, mean [SD] 12.2 [1.8] years). Twelve patients with premenarche status at baseline had not reached menarche by end of study (age ranged from 10 to 13 years at end of study), in any of the pooled studies. Among all patients who were postmenarche at baseline or during the study (n = 92), 27 (29.3%) reported experiencing an amenorrhea event. Seventeen of these patients (18.5%) experienced a single occurrence, and 15 patients (16.3%) had amenorrhea that lasted > 6 months (Table 3). When only the longest duration period of amenorrhea for each patient was considered, the median duration was 291 days, and most events resolved without intervention. A total of 2 dose interruptions/reductions were ordered to manage amenorrhea events. In addition, 19 of 92 patients (20.7%) who were postmenarche at baseline or during the study experienced irregular menstruation; these were all experienced as a single irregular menstruation event. In 12 patients (13.0%), the event lasted > 6 months (Table 3). The median duration of irregular menstruation events was 277 days. A total of 4 dose interruptions/reductions were ordered to manage irregular or delayed menstruation (oligomenorrhea). No menstrual irregularity events at any stage led to permanent discontinuation of study drug.

**Table 3:** Menstrual irregularities in postmenarche patients on everolimus.

n (%)	Everolimus n = 92	
	Amenorrhea	Irregular Menstruation
**At least 1 event, n (%)**	27 (29.3)	19 (20.7)
**Duration of event, n (%)**^**a**^		
** 3 months or less**	8 (8.7)	5 (5.4)
** > 3 to 6 months**	4 (4.3)	2 (2.2)
** > 6 months**	15 (16.3)^b^	12 (13.0)
**Duration of longest event,**^**c**^ **days**		
** Mean (SD)**	295.7 (246.3)	394.3 (414.5)
** Median (range)**	291.0 (10–933)	277 (1–1373)

SD, standard deviation.

^a^A patient who experienced multiple events of irregular menstruation while being treated is only counted under the duration of the longest event.

^b^Classified as amenorrhea per National Cancer Institute Common Terminology Criteria for Adverse Events version 4.0.

^c^Longest event for each patient with 1 or more events.

**Fig 4:**
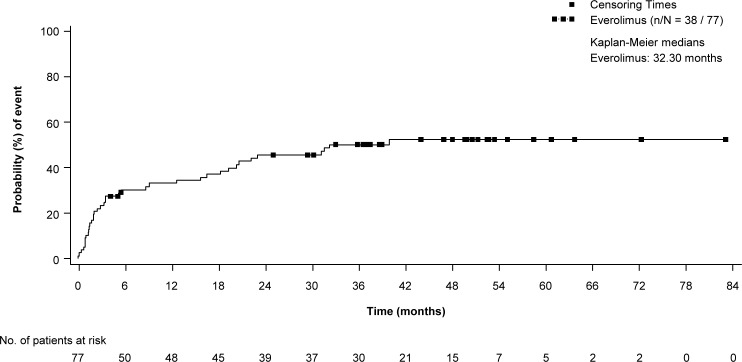
Time to onset of menstrual irregularities in patients who were postmenarche at baseline.

The first menstrual related event occurred within 6 months of initiating everolimus in 26 of the 84 patients (30%) who were postmenarche at baseline. The onset of a substantial number of events occurred years later, reaching a plateau after 3 years of treatment (Fig 4).

The majority of amenorrhea events were grade 1 or 2 and resolved without intervention. Among the 7 patients with grade 3/4 amenorrhea, 5 instances resolved spontaneously without intervention (duration, 215–642 days), 1 resolved with concomitant medication (progesterone 100 mg QD; duration of event, 296 days), and 1 was ongoing at the end of the study (duration, > 774 days). Two additional patients reported experiencing amenorrhea > 1 year that had not resolved by study completion; these events lasted > 435 days and > 933 days. The patient with amenorrhea lasting > 933 days was a 34-year-old woman using hormonal contraception, with a medical history of hypothyroidism.

### Endocrine function

LH and FSH levels were monitored in the 3 studies. Increased LH level was reported as an adverse event in 3 patients. However, further review of these 3 cases showed the levels to be within the normal physiological levels of LH surge at the start of the reported event (range, 21–24 IU/L). FSH levels were also within normal physiological limits for these patients. No trends or patterns were observed in the reproductive endocrinology laboratory results of the pooled population.

## Discussion

The results of this study add to the growing body of knowledge regarding the safety of everolimus in patients with TSC. These findings demonstrated that 38.4% of the female patient population who received at least 1 dose of everolimus experienced fertility-related or menstrual irregularity events. The most frequent events were amenorrhea and irregular menstruation. Although these events were common (24% and 17%, respectively), the majority resolved without intervention. Six events resolved promptly following everolimus dose modifications. In addition, everolimus was associated with more fertility- or menstruation-related events in the double-blind phases compared with placebo. Interestingly, however, long-term treatment with everolimus did not appear to affect sexual maturation in these patients, as evidenced by the age of menarche during treatment in the current analyses. These data provide valuable insight into the effects of mTOR inhibitors in female patients of child-bearing potential.

Observations of mTOR signaling have suggested it to have a potential role as a metabolic gauge and as a mediator for the effects of leptin on food intake and energy homeostasis [15]. Caloric restriction, which is known to reduce mTOR activity in a wide variety of cell types and conditions, is associated with suppression of ovarian follicular development in adult female rats [21]. In line with this, mTOR signaling may thus also play a role in the transmission of metabolic information to the reproductive centers involved in the onset of puberty [15]. Links between mTOR activation and the actions of FSH via the PI-3-kinase/AKT pathway have also been demonstrated in previous studies [22]. Furthermore, reports evaluating steroidogenesis regulation in granulosa lutein cells have identified an mTOR-associated role [23]. Although steroidogenesis has been observed to be affected by mTOR inhibitors, the degree of impact is unknown [24]. In consideration of this, the use of an mTOR inhibitor, such as everolimus, may affect fertility and reproductive events in female patients with TSC or in other diseases requiring mTOR inhibitor-based therapies. In addition, a retrospective analysis of sirolimus and everolimus treatment in men revealed decreases in testosterone levels, and increases in FSH and LH levels [24,25]. Investigation into other everolimus trials regarding fertility events, in non-TSC patient groups, may be an interesting extension of the current analysis; however, it is possible that these events were not reported as the patient populations in renal cell carcinoma and neuroendocrine tumor studies are typically older in age.

There were some potential limitations of this current study. Firstly, interpretation of the findings of this pooled analysis may be complicated by the heterogeneity of the study population, which included patients from a broad age group (13.4% aged <10 years and 6.7% aged 46–55 years at baseline), different dosing regimens used in the studies (weight-based dosing in patients with SEGA, 10 mg/day in patients with renal angiomyolipoma), and a variable number of doses received. Secondly, dose-response was not assessed, and it may be that patients on higher doses experienced more or longer events. Finally, according to the CTCAE, the definition of amenorrhea is the absence of menses for more than 6 months [26]; however, 12 patients with absence of menses less than 6 months were coded by investigators as having had amenorrhea, and 12 patients were coded as having irregular menses (but not amenorrhea) for more than 6 months, reflecting those patients who presented with an irregular cycle but without the complete absence of menses. As highlighted here, the potential for errors in coding are a limitation of this current analysis. In addition, baseline values for many patients were not available and therefore the interpretation of some results presented a challenge.

Investigators for future studies may wish to examine if any changes in menstruation are dose-related effects, and if this is a class effect of all mTOR inhibitors. In addition, potential implications on longer-term fertility and any impact on childbearing potential in female patients may be of interest.

## Conclusions

This pooled analysis suggests that treatment with everolimus for up to 6.5 years is generally well tolerated regarding endocrine function and menstrual irregularities in female patients with TSC-associated SEGA or angiomyolipoma. Everolimus did not adversely affect menarche. Secondary amenorrhea and irregular menstruation were the main fertility-related adverse events, and most resolved without intervention. The fertility events that occurred were mostly mild and transient, and everolimus did not seem to impact hormonal levels.

## Supporting information

S1 Table List of ethics committees and/or intuitional review boards that approved the EXIST-2 study. (DOCX)
