# Supplementary material for: Pooled analysis of menstrual irregularities from three major clinical studies evaluating everolimus for the treatment of tuberous sclerosis complex
Source: PLoS One. 2017 Oct 12;12(10):e0186235. doi: 10.1371/journal.pone.0186235 (PMC5638404; doi:10.1371/journal.pone.0186235)
Supplement: S1 Table — (DOCX) [file pone.0186235.s001.docx]

**S1 Table**.

The Phase 2 study (NCT00411619) was conducted at the Cincinnati Children’s Hospital Medical Center Tuberous Sclerosis Clinic. The IRB is listed below.

| **Ethics Committee or**  **Institutional Review Board** | **Department /**  **Organization** | **City, State/Province,**  **Postal Code**  **Country** |
| --- | --- | --- |
| Institutional Review Board | Cincinnati Children's  Hospital Medical center | Cincinnati MLC 5020, United States |

List of Independent Ethics Committees (IEC) or Institutional Review Boards (IRB) by study center for EXIST-1 (NCT00789828)

| **Center No.** | **Ethics Committee or**  **Institutional Review Board** | **Department /**  **Organization** | **City, State/Province,**  **Postal Code**  **Country** |
| --- | --- | --- | --- |
| 0100 | SESIAHS Human Research  Ethics Committee | SESIAHS Northern  Hospital Network -  Research Office | Randwick NSW 2130,  Australia |
| 0150 | Commissie voor Medische Ethiek | Universitair Ziekenhuis Brussel | Brussel 1090, Belgium |
| 0250 | Research Ethics Board | The Sick Childrens Hospital | Toronto M5G 1X8, Canada |
| 0251 | Comité d'éthique de recherche | Hôpital Saint Justine | Montreal Quebec H3T,  IC5  Canada |
| 0352 | Ethik-Kommission | Medizinischen Falultät Heidelberg | Heidelberg 69115,  Germany |
| 0353 | Geschäftsstelle der EthikKommission des Landes Berlin | Landesamt für  Gesundheit und Soziales | Berlin 10707,  Germany |
| 0400 | Comitato Etico Indipendente | Azienda Ospedaliera  Universitaria Policlinico  Tor Vergata di Roma | Roma 00133,  Italy |
| 0403 | Comitato Di Etica | IRCSS Istituto Giannina Gaslini di Genova | Genova GE 16147, Italy |
| 0450 | Medische Ethische Toetsingscommissie | Universitair Medisch  Centrum Utrecht | Utrecht 3508 GA,  Netherlands |
| 0600 | Komisja Bioetyczna | Instytucie "Pomnik  Zdrowia Dziecka" | Warszawa 04-730,  Poland |
| 0700 | Ethics Committee | Moscow research institute of pediatrics and children surgery, Ministry of Health of Russia | Moscow NA 125412, Russia |
| 0753 | Southampton and South West Hampshire Research Ethics  Committee | NHS - Health Research Authority | Southampton Hampshire  SO16 4RJ,  United Kingdom |
| 0500 | Institutional Review Board | Cincinnati Children's  Hospital Medical center | Cincinnati MLC 5020, United States |
| 0501 | Western Institutional Review Board | WIRB - Copernicus Group | Olympia WA 98502, United States |
| 0502 | Western Institutional Review Board | WIRB - Copernicus Group | Olympia WA 98502, United States |
| 0503 | 1. Adult consent: Institutional Review Board 2. Children consent: Institutional Review Board | 1. Allina Health System 2. Children's Hospitals and Clinics of Minnesota | Minneapolis MN 55404, United States  Minneapolis MN 55404, United States |
| 0504 | Partners Human Research office | Partners HealthCare | Boston MA 02116, United States |
| 0509 | Institutional Review Board | The University of Chicago | Chicago IL 60637, United States |
| 0510 | Institutional Review Board | The University of Texas, Southwestern medical center | Dallas TX 75390-8843,  United States |
| 0511 | Western Institutional Review Board | WIRB - Copernicus Group | Olympia WA 98502, United States |
| 0512 | Office of Human Research Studies | Dana Farber Cancer Institute | Boston MA 02115, United States |
| 0514 | Internal Review Board | St. Joseph's Hospital and Medical Center | Phoenix AZ 85013, United States |
| 0515 | Institutional Review Board | Children's Hospital and Research Center Oakland | Oakland CA 94609 United States |
| 0516 | Institutional Review Board | Children's Healthcare of Atlanta | Atlanta GA 30329, United States |

List of Independent Ethics Committees (IEC) or Institutional Review Boards (IRB) by study center for EXIST-2 (NCT00790400)

| **Center No.** | **Ethics Committee or**  **Institutional Review Board** | **Department /**  **Organization** | **City, State/Province,**  **Postal Code**  **Country** |
| --- | --- | --- | --- |
| 0100 | Research Ethics Board | University Health Network | Ontario M5G 1Z5  Canada |
| 0154 | Institutional Review Board | CPP "Sud-Est IV" LYON  Centre Régional de Lutte  Contre le cancer Léon  Bérard | Lyon 69373  France |
| 0201 | Institutional Review Board | Landesamt für Gesundheit  und Soziales  Ethik-Kommission des  Landes | Berlin 10707  Germany |
| 0202 | Institutional Review Board | Ludwig-Maximilians-  Universität München  Klinikum der Universität  Ethik-Kommission | Munchen 80336  Germany |
| 0251 | Ethics Committee | Comitato Indipendente  presso la Fondazione PTV  Policlinico Tor Vergata Di  Roma Viale | Roma 00133  Italy |
| 0253 | Ethics Committee | Comitato Etico  Interaziendale A.O. Citta'  Della Salute E Della Scienza | Torino 10126  Italy |
| 0254 | Ethics Committee | Comitato Etico Locale Per  La Sperimentazione Clinica  Dei Medicinali Dell’Azienda  Ospedaliera Universitaria  Senese Di Siena c/o UOC  Farmacia AOUS - Viale  Bracci | SIENA 53100  Italy |
| 0300 | Institutional Review Board | METC UMCU  Huispost D 01.343 | 3584 CX Utrecht  Netherlands |
| 0400 | Institutional Review Board | Moscow Federal State  Budget Scientific Institution  of Pediatrics and Pediatrics  Surgery | Moscow 127412  Russia |
| 0476 | Ethics Committee | The Bioethics Committee at Instytut, Pomnik- Centrum  Zdrowia Dziecka” | Warszawa 04-730  Poland |
| 0477 | Ethics Committee | The Bioethics Committee at Instytut, Pomnik- Centrum  Zdrowia Dziecka” | Warszawa 04-730  Poland |
| 0550 | Institutional Review Board | Cincinnati Children’s  Hospital Medical Center | Cincinnati OH 45229  USA |
| 0551 | Western Institution Review  Board (WIRB) |  | Seattle, Washington 98502  USA |
| 0552 | Institutional Review Board | Shulman Associates IRB | Lake Forest Dr Blue Ash 45242  (513) 761-4100  USA |
| 0553 | Institutional Review Board | Partners Human Research  Office | Boston 02116 |
| 0555 | Institutional Review Board | St. Joseph's Hospital and  Medical Center | Phoenix, AZ 85013 |
| 0556 | Institutional Review Board | University of Tennessee | Memphis  United States |
| 0601 | Institutional Review Board | Hokkaido University Hospital | Hokkaido  Japan |
| 0602 | Ethics Committee or  Institutional Review Board | Yamagata university hospital | Yamagata  Japan |
| 0603 | Institutional Review Board | Osaka University Hospital | Osaka 565-0871  Japan |
| 0650 | Ethics Committee | Comité Etico De  Investigacion Clinica  Fundacio Puigvert  Coneixement | Barcelona 08025 |
| 0770 | Institutional Review Board | South Central –  Southampton A  South West REC Centre | Bristol BS1 2NT  UK |
| 0772 | Institutional Review Board | South Central –  Southampton A  South West REC Centre | Bristol BS1 2NT  UK |
| 0774 | Institutional Review Board | South Central –  Southampton A  South West REC Centre | Bristol BS1 2NT  UK |
| 0775 | Institutional Review Board | South Central –  Southampton A  South West REC Centre | Bristol BS1 2NT  UK |
